# Ulcer Perforation on Excluded Stomach Following Gastric Bypass Surgery: A Case Report of Atypical Presentation Without Pneumoperitoneum

**DOI:** 10.7759/cureus.98785

**Published:** 2025-12-09

**Authors:** Nuno Gonçalves, José Pedro Pinto, Ana Ribeiro, Fernando Manso, Joaquim M Costa Pereira

**Affiliations:** 1 School of Medicine, University of Minho, Braga, PRT; 2 General Surgery Department, Hospital de Braga, Braga, PRT; 3 General Surgery, Unidade Local de Saúde de Braga, Braga, PRT; 4 Bariatric Surgery, Unidade Local de Saúde de Braga, Braga, PRT; 5 Colorectal Surgery, Unidade Local de Saúde de Braga, Braga, PRT

**Keywords:** bariatric surgery complications, gastric bypass surgery, gastric ulcer perforation, minimally invasive surgery, pneumoperitoneum

## Abstract

Roux-en-Y gastric bypass (RYGB) is widely used to treat morbid obesity, but it may lead to rare complications involving the excluded stomach. Ulcers in this defunctionalized segment are particularly uncommon yet clinically significant due to their potential to perforate and present atypically. Gastric perforation following RYGB is a rare but potentially fatal complication. This case report describes a 41-year-old patient who presented with gastric perforation without the typical diagnostic marker of pneumoperitoneum. Through comprehensive clinical evaluation, laboratory testing, and imaging, this report highlights the importance of thorough diagnostic approaches in post-bariatric surgery patients, emphasizing the necessity of a low threshold for surgical intervention.

## Introduction

Bariatric surgery has become increasingly common in the management of morbid obesity, providing significant weight loss and improvement in obesity-related comorbidities [1-3]. However, as the population of bariatric surgery patients grows, so does the recognition of rare but serious complications, including gastric perforation. The incidence of gastric perforation after gastric bypass surgery, particularly at the anastomosis between the gastric pouch and the jejunum, has been reported to be less than 5%. Ulcers in the excluded stomach, also known as remnant gastric ulcers, represent a rare but critical complication following Roux-en-Y gastric bypass (RYGB) surgery. In RYGB, the gastric remnant and duodenum are excluded from the alimentary tract, making endoscopic access to this segment difficult and contributing to delays in diagnosing ulcers or perforations in the defunctionalized pouch. Unlike marginal ulcers, which occur at the gastrojejunostomy, excluded stomach ulcers develop in the remnant gastric pouch that has been bypassed and no longer participates in food digestion. These ulcers pose a considerable risk of morbidity and mortality if not diagnosed promptly and accurately [4-6].

The pathophysiology of these ulcers is complex and multifactorial, involving several predisposing factors such as Helicobacter pylori infection, smoking, the use of non-steroidal anti-inflammatory drugs (NSAIDs), and the size of the gastric pouch. Although the excluded stomach is bypassed from the normal alimentary flow, acid secretion persists because the parietal cells remain physiologically active. In the absence of food intake through this segment, gastric secretions are not buffered, creating a more acidic environment that can predispose to mucosal injury. Additionally, stasis of gastric fluids within the defunctionalized pouch may contribute to localized irritation and chemical inflammation. Altered vagal stimulation after RYGB can further disrupt mucosal defense mechanisms, while microvascular ischemia resulting from changes in blood supply may impair tissue resilience and delay healing. Some authors have also proposed that exposure to bile or pancreatic secretions may exacerbate mucosal vulnerability in the remnant stomach. These physiological alterations, together with patient-related factors such as smoking, NSAID use, and H. pylori infection, help explain why ulceration can develop in a segment that no longer participates in digestion and why its progression to perforation may occur insidiously. These factors heighten the risk of perforation, making an understanding of these mechanisms critical for effective prevention strategies. Excluded stomach ulcers are even rarer, with reported incidences ranging from approximately 0.12% to 0.84% [5,7,8]. These ulcers can lead to significant complications, including perforation and bleeding, often requiring urgent surgical intervention [9,10]. Although diminished, gastric acid production continues after gastric bypass, contributing to the potential for ulceration.

Gastric perforation typically presents with a classic triad of symptoms: sudden onset of abdominal pain, peritoneal signs, and sometimes fever. In most cases, the presence of pneumoperitoneum, indicative of free air in the abdominal cavity, serves as a critical diagnostic marker for perforation. However, this classic presentation can be misleading, as the absence of pneumoperitoneum does not preclude the existence of a perforation, particularly in patients with a history of prior gastric surgery, where reduced or even absent air in the bypassed stomach may occur. Patients often report only epigastric or upper abdominal pain.

Previous literature indicates that the absence of pneumoperitoneum may lead to misdiagnosis or delayed treatment, with practitioners potentially attributing acute abdominal symptoms to other, less critical conditions. Negative X-ray or CT findings do not exclude the possibility of internal organ perforation in these patients. This case report aims to underscore the importance of a comprehensive clinical evaluation, including the assessment of elevated inflammatory markers, peritoneal effusion on imaging studies, and a high index of suspicion for internal hernias or other surgical complications in patients with a history of bariatric surgery. As bariatric surgery continues to gain popularity, healthcare providers must recognize that these complications, though infrequent, can have devastating effects if not identified and treated promptly. This case highlights the necessity of considering alternative diagnostic markers, such as laboratory abnormalities and imaging findings, to avoid delays in management.

## Case presentation

This case report presents a detailed evaluation of a 41-year-old male patient with a history of gastric bypass surgery for morbid obesity six years prior. He was admitted to the emergency department with acute abdominal symptoms characterized by generalized, intense, colicky pain that worsened in the supine position and during trunk flexion. The patient reported nausea but denied vomiting, fever, or other significant symptoms.

Physical examination revealed diaphoresis and a soft, depressible abdomen, although voluntary contraction of the rectus abdominis muscle complicated the assessment of peritoneal irritation signs. Laboratory tests indicated leukocytosis (19.3 × 10^3^) with neutrophilia (92.4%), elevated amylase (497 U/L), lipase (164 U/L), and C-reactive protein (140 mg/L).

**Figure 1:**
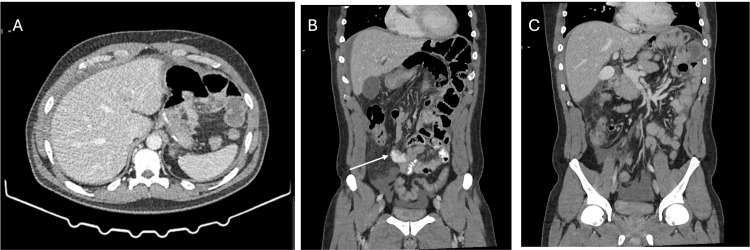
Contrast-enhanced abdominal and pelvic CT scan. Axial (A) and coronal (B and C) views demonstrating perihepatic, paracolic, and pelvic free fluid without evidence of pneumoperitoneum or pneumatosis intestinalis. A subtle mesenteric “swirl sign” is visible (arrow), raising suspicion for an internal hernia and prompting surgical exploration.

Contrast-enhanced CT (Figure 1) of the abdomen and pelvis revealed peritoneal effusion in the perihepatic area, right paracolic gutter, and pelvic cavity, with no evidence of pneumoperitoneum or pneumatosis intestinalis. However, the imaging did show a swirl sign of vessels, which raised suspicion for an internal hernia, prompting further surgical evaluation. Based on the clinical suspicion, laboratory findings, and imaging results, the decision was made to proceed with exploratory laparoscopy.

**Figure 2:**
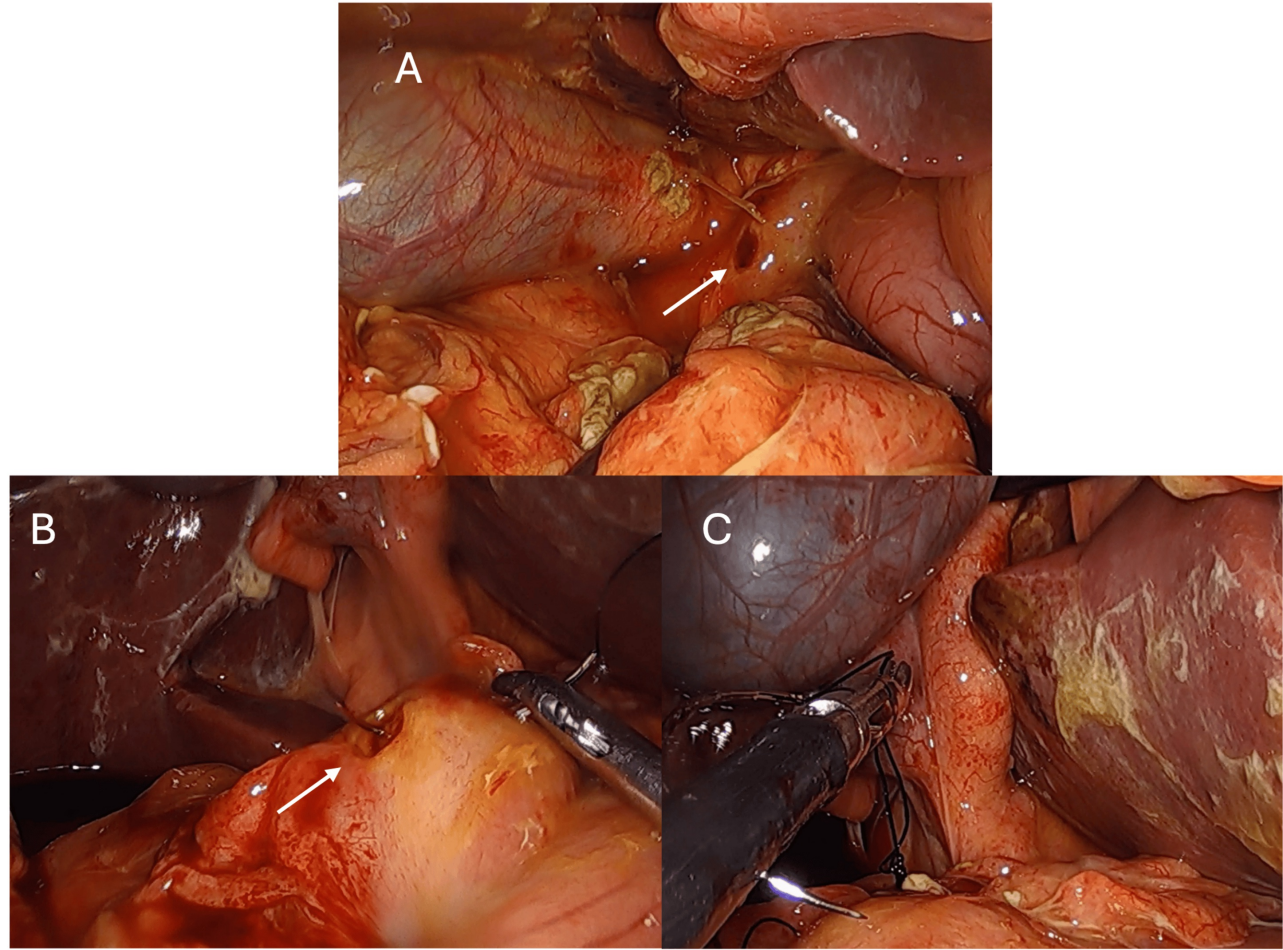
Intraoperative laparoscopic findings. (A) Bilious peritoneal effusion and inflammatory changes surrounding the excluded stomach. (B) Identification of a pre-pyloric perforated gastric ulcer (arrows) with active leakage. (C) Laparoscopic suture repair of the perforation.

The exploratory procedure (Figure 2) revealed biliary peritonitis with inflammatory changes surrounding the excluded stomach. A pre-pyloric perforated ulcer was identified, with visible leakage of bile. Laparoscopic suture repair was performed successfully. The patient experienced an uneventful postoperative course and was discharged without complications on the 6th postoperative day. An intraoperative biopsy was obtained, and histopathology confirmed the absence of malignancy. At the 12-month clinical follow-up, the patient remained asymptomatic with no recurrence of symptoms, maintaining daily IBP.

## Discussion

The presence of ulcers in the excluded stomach presents unique clinical challenges. Patients may exhibit atypical symptoms, often limited to vague abdominal discomfort rather than the classical signs of acute abdomen [8,9]. This atypical symptomatology can lead to delays in diagnosis and treatment, which are crucial given the high risk of complications such as perforation. Clinicians must maintain a high index of suspicion for these ulcers, particularly in patients with a history of RYGB who present with unexplained abdominal symptoms.

An important and atypical aspect of this case is the absence of pneumoperitoneum on CT imaging, despite the presence of a clear perforation. This can be explained by several physiological and anatomical factors unique to post-RYGB patients. Because the excluded stomach no longer receives swallowed air or ingested material, the intraluminal gas content is minimal, which significantly reduces the likelihood of detecting free air even when a perforation occurs. Furthermore, the pre-pyloric location of the ulcer may promote a contained leak, as this region is closely surrounded by adjacent structures that can rapidly limit the escape of air. Early sealing by inflamed tissue or the omentum may also prevent the accumulation of detectable intraperitoneal gas. As a result, perforations of the remnant stomach may present predominantly with fluid collections or inflammatory changes rather than free air, underscoring that a normal CT scan cannot reliably exclude perforation in post-RYGB patients.

The absence of pneumoperitoneum complicates diagnosis and may contribute to delays in treatment. In such cases, a comprehensive clinical evaluation becomes essential, particularly when laboratory abnormalities, such as elevated inflammatory markers, and imaging findings of peritoneal effusion raise concern for an underlying surgical emergency. In this patient, although the suspected diagnosis of an internal hernia was not confirmed, the constellation of findings warranted early surgical exploration, which ultimately identified and treated the perforation.

Given the patient’s history of RYGB and the severity of his symptoms, several differential diagnoses were considered, including internal hernia, acute pancreatitis, perforation of another viscus, and complicated marginal ulcer disease. Although imaging demonstrated peritoneal effusion and a mesenteric swirl sign, it did not provide a definitive diagnosis, and the absence of pneumoperitoneum further contributed to diagnostic uncertainty. In post-bypass patients, the altered anatomy often limits the accuracy of both clinical examination and radiologic interpretation, making sole reliance on imaging insufficient. Therefore, the combination of persistent abdominal pain, markedly elevated inflammatory markers, and equivocal CT findings justified proceeding with exploratory laparoscopy. This approach remains essential in high-risk post-RYGB patients, as early operative evaluation can prevent delays in identifying life-threatening complications.

A review of the literature reveals a small but significant number of reported cases of perforated ulcers in the excluded stomach. For instance, previous studies have documented that a majority of patients with perforated ulcers present without prior warning signs, underscoring the unpredictable nature of this complication [7-9].

Management of perforated ulcers in the excluded stomach typically involves surgical intervention, such as laparoscopic repair with omental patching or subtotal gastrectomy when necessary [8,10]. Postoperatively, empirical H. pylori treatment and long-term proton pump inhibitor therapy are recommended to prevent recurrence of ulceration [10].

Moreover, this case illustrates the importance of interdisciplinary collaboration in managing complex post-bariatric patients. The decision to proceed with exploratory laparoscopy resulted from a careful synthesis of the patient’s clinical history, laboratory abnormalities, and imaging findings, highlighting the need for an individualized and comprehensive approach. Early surgical exploration is particularly critical in this population, as diagnostic delays can substantially increase morbidity and mortality.

In addition, the case reinforces that the absence of pneumoperitoneum on CT does not exclude the possibility of remnant stomach perforation. Because the excluded stomach contains minimal intraluminal air and perforations may be anatomically contained, clinicians should instead prioritize warning signs such as peritoneal effusion, elevated inflammatory markers, and persistent abdominal pain. Given the limitations of imaging and endoscopic access in post-RYGB anatomy, maintaining a low threshold for exploratory laparoscopy is essential to ensure timely diagnosis and prevent complications.

## Conclusions

In conclusion, ulcers in the excluded stomach following RYGB are a significant yet often overlooked complication. Clinicians should be aware of the unique risks associated with these ulcers, including their potential to cause severe morbidity due to perforation. Continuous monitoring and a high index of suspicion are essential for early diagnosis and management. Additionally, adopting standardized protocols for postoperative care, including the use of proton pump inhibitors and careful evaluation of abdominal symptoms, can mitigate the risks associated with this serious condition. As the number of bariatric surgeries continues to rise, enhancing our understanding of excluded stomach ulcers will be vital in improving patient safety and outcomes.

This case reinforces the necessity for a low threshold for intervention in post-bariatric surgery patients. Early identification and management of such complications are vital in preventing more severe peritoneal and systemic consequences, ensuring optimal patient outcomes.
